# The effect of active visual art therapy on health outcomes: protocol of a systematic review of randomised controlled trials

**DOI:** 10.1186/s13643-022-01976-7

**Published:** 2022-05-16

**Authors:** Ronja Joschko, Stephanie Roll, Stefan N. Willich, Anne Berghöfer

**Affiliations:** Institute for Social Medicine, Epidemiology and Health Economics, Charité – Universitätsmedizin Berlin, corporate member of Freie Universität Berlin and Humboldt-Universität zu Berlin, Luisenstr. 57, 10117 Berlin, Germany

**Keywords:** Active visual art therapy, Creative therapy, Therapy effectiveness evaluation, Systematic review, Mental health, Psychosomatic disorders, Depression, Anxiety, Quality of life

## Abstract

**Background:**

Art therapy is a form of complementary therapy to treat a wide variety of health problems. Existing studies examining the effects of art therapy differ substantially regarding content and setting of the intervention, as well as their included populations, outcomes, and methodology. The aim of this review is to evaluate the overall effectiveness of active visual art therapy, used across different treatment indications and settings, on various patient outcomes.

**Methods:**

We will include randomised controlled studies with an active art therapy intervention, defined as any form of creative expression involving a medium (such as paint etc.) to be actively applied or shaped by the patient in an artistic or expressive form, compared to any type of control. Any treatment indication and patient group will be included. A systematic literature search of the Cochrane Library, EMBASE (via Ovid), MEDLINE (via Ovid), CINAHL, ERIC, APA PsycArticles, APA PsycInfo, and PSYNDEX (all via EBSCOHost), ClinicalTrials.gov and the WHO’s International Clinical Trials Registry Platform (ICTRP) will be conducted. Psychological, cognitive, somatic and economic outcomes will be used. Based on the number, quality and outcome heterogeneity of the selected studies, a meta-analysis might be conducted, or the data synthesis will be performed narratively only. Heterogeneity will be assessed by calculating the p-value for the chi^2^ test and the *I*^2^ statistic. Subgroup analyses and meta-regressions are planned.

**Discussion:**

This systematic review will provide a concise overview of current knowledge of the effectiveness of art therapy. Results have the potential to (1) inform existing treatment guidelines and clinical practice decisions, (2) provide insights to the therapy’s mechanism of change, and (3) generate hypothesis that can serve as a starting point for future randomised controlled studies.

**Systematic review registration:**

PROSPERO ID CRD42021233272

**Supplementary Information:**

The online version contains supplementary material available at 10.1186/s13643-022-01976-7.

## Background

**Fig. 1:**
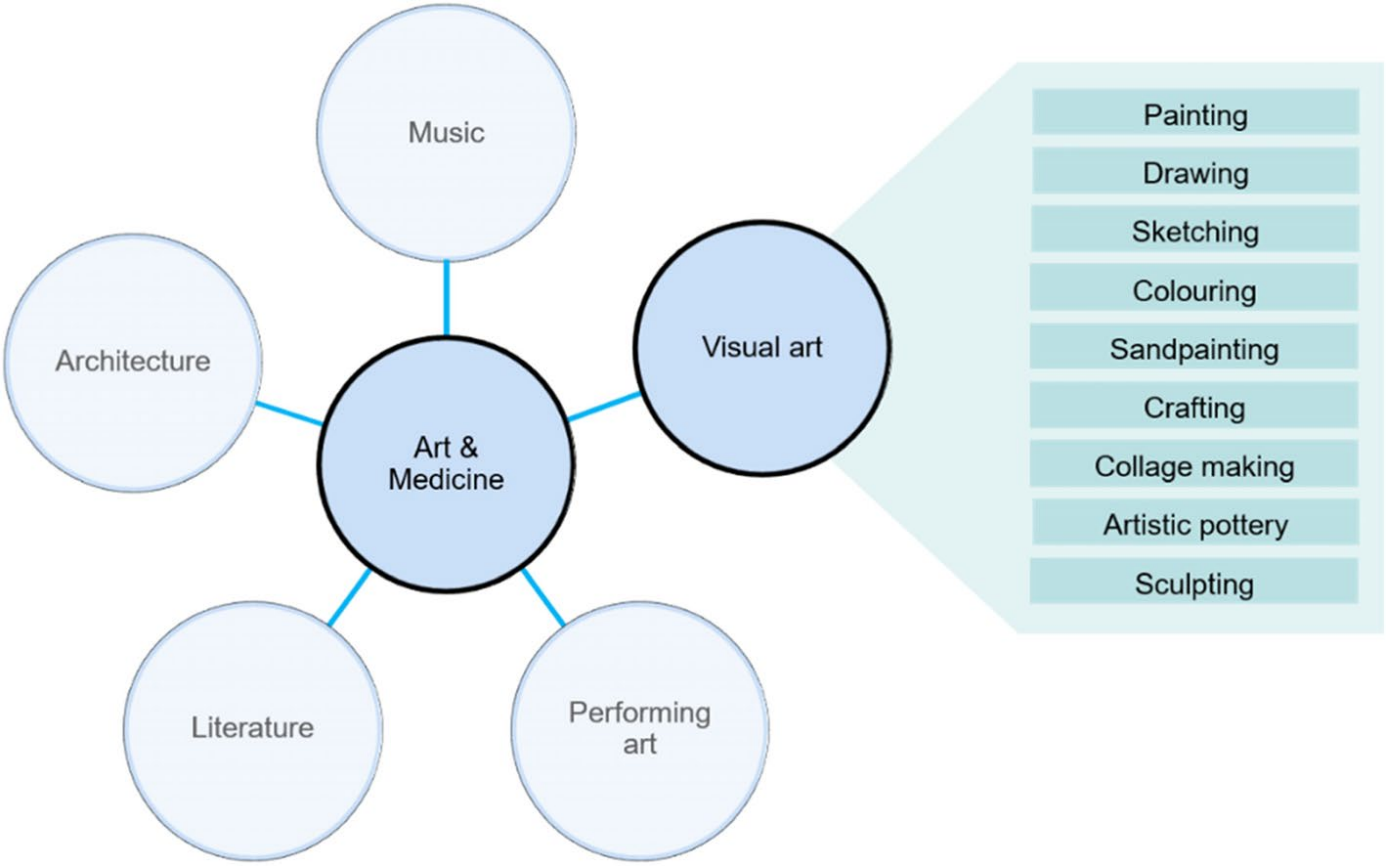
The five clusters of art used in medicine for therapeutic purposes, with examples of active visual art forms (figure created by the authors)

Complementary and integrative treatment methods can play an important role when treating various chronic conditions. Complementary medicine describes treatment methods that are added to the standard therapy regiment, thereby creating an integrative health approach, in the anticipation of better treatment effects and improved health outcomes [1]. Within a broad field of therapeutic approaches that are used complementarily, art therapy has long occupied a wide space. After an extensive sighting of the literature, we decided to differentiate between five clusters of art that are used in combination with standard therapies: visual arts, performing arts, music, literature, and architecture (Fig. 1). Each cluster can either be used actively or receptively.

Active visual art therapy (AVAT) is often used as a complementary therapy method, both in acute medicine and in rehabilitation. The use of AVAT is frequently associated with the treatment of psychiatric, psychosomatic, psychological, or neurological disorders, such as anxiety [2], depression [3], eating disorders [4], trauma [5, 6], cognitive impairment, or dementia [7]. However, the application of AVAT extends beyond that, thereby broadening its potential benefits: it is also used to complement the treatment of cystic fibrosis [8] or cancer [9, 10], to build up resilience and well-being [11, 12], or to stop adolescents from smoking [13].

As a complementary intervention, AVAT aims at reducing symptom burden beyond the effect of the standard treatment alone. Since AVAT is thought to be side effect free [14] it could be a valuable addition to the standard treatment, offering symptom reduction with no increased risk of adverse events, as well as an potential improvement in quality of life [15–17].

The existing literature examining the effectiveness of art therapy has shown some positive results across a wide variety of treatment indications, such as the treatment of depression [3, 18], anxiety [19, 20], psychosis [21], the enhancement of mental wellbeing [22], and the complementary treatment of cancer [15, 23]. However, the existing evidence is characterised by conflicting results. While some studies report favourable results and treatment successes through AVAT [17, 24–26], many studies report mixed results [3, 15, 16, 27, 28]. There is a substantial number of systematic reviews which examine the effectiveness of art therapy regarding individual outcomes, such as trauma [29–33], anxiety [19] mental health in people who have cancer [23, 34, 35] dementia [7], and potential harms and benefits of the intervention [36]. The limited number of published studies, however, can make the creation of a systematic review difficult, especially when narrowing down additional factors, such as the desired study design [7].

Therefore, it might be helpful to combine all existing evidence on the therapeutic effects of AVAT in one review, to generate evidence regarding its overall effectiveness. To our knowledge, there is no systematic review that accumulates the data of all published RCTs on the topic of AVAT, while abiding to strict methodological standards, such as the Cochrane handbook [37] and the PRISMA statement [38]. We thus aim to establish and strengthen the existing evidence basis for AVAT, reflecting the clinical reality by including a wide variety of settings, populations, and treatment indications. Furthermore, we will try to identify characteristics of the setting and the intervention that may increase AVAT’s effectiveness, as well as differences in treatment success for different conditions or reasons for treatment.

## Methods/Design

### Registration and reporting

We have submitted the protocol to PROSPERO (the International Prospective Register of Systematic Reviews) on February 9, 2021 (PROSPERO ID: CRD42021233272). In the writing of this protocol we have adhered to the adapted PRISMA-P (Preferred reporting items for systematic review and meta-analysis protocols, see Additional file 1) [39]. Important protocol amendments will be submitted to PROSPERO.

### Eligibility criteria

#### Type of study

We will include randomised controlled trials to minimise the sources of bias possibly arising from observational study designs.

#### Types of participants

As AVAT is used across many patient populations and settings, we will include patients across all treatment indications. Thus, we will include populations receiving curative, palliative, rehabilitative, or preventive care for a variety of reasons. Patients of all ages (including seniors, children and adolescents), all cultural backgrounds, and all living situations (inpatients, outpatients, prison, nursing homes etc.) will be included without further restrictions. The resulting diversity reflects the current treatment reality. Heterogeneity of included studies will be accounted for by subgroup analyses at the stage of data synthesis. Differences in treatment success depending on population characteristics are furthermore of special interest in this review.

#### Types of interventions

As the therapeutic mechanisms of AVAT are not yet unanimously agreed upon, we want to reduce the heterogeneity of treatment methods included by focusing on only one cluster of art activities (active visual art).

We define AVAT as any form of creative expression involving a medium such as paint, wax, charcoal, graphite, or any other form of colour pigments, clay, sand, or other materials that are applied or shaped by the individual in an artistic or expressive form.

The interventions must include a therapeutic element, such as the targeted guidance from an art therapist or a reflective element. Both, group and individual treatment in any setting are included.

Purely occupational activities not intended to have a therapeutic effect will not be considered.

All forms of music, dance, and performing art therapies, as well as poetry therapy and (expressive) writing interventions which focus on the content rather than appearance (like journal therapy) will not be included. Studies with mixed interventions will be included only if the effects of the AVAT can be separated from the effects of the other treatments. Furthermore, all passive forms of visual art therapy will be excluded, such as receptive viewings of paintings or pictures.

#### Comparison interventions

Depending on the treatment indication and setting, the control group design will likely vary. We will include studies with any type of control group, because art therapy research, just like psychotherapy research, must face the problem that there are usually no standard controls like, e.g. a placebo [40]. Therefore, we will include all control groups using treatment as usual (including usual care, standard of care etc.), no treatment (with or without waitlist control design), or any active control other than AVAT (such as attention placebo controls) as potential comparators.

### Stakeholder involvement

Stakeholders will be involved to increase the relevance of the study design. Patients, art therapists, and physicians prescribing art therapy, all from a centre that uses AVAT regularly, will be interviewed using a semi structured questionnaire that captures the expert’s perspective on meaningful outcomes. Particularly, we are interested in the stakeholders’ opinions about which outcomes might be most affected by AVAT, which individual differences might be expected, and which other factors could affect the effectiveness of AVAT.

A second session might be held at the stage of result interpretation as the stakeholders’ perspective could be a valuable tool to make sense of the data.

### Outcomes

As there is no universal standard regarding the outcomes of AVAT, we have based our choice of outcome measures on selected, high quality work on the subject [7], and on theoretical considerations.

Outcome measures will include general and disease specific quality of life, anxiety, depression, treatment satisfaction, adverse effects, health economic factors, and other disorder specific outcomes. The latter are of special relevance for the patients and have the potential to reflect the effectiveness of the therapy. The disorder specific outcomes will be further clustered into groups, such as treatment success, mental state, affect and psychological wellbeing, cognitive function, pain (medication), somatic effects, therapy compliance, and motivation/agency/autonomy regarding the underlying disease or its consequences. Depending on the included studies, we might re-evaluate these categories and modify the clusters if necessary.

Outcomes will be grouped into short-term and long-term outcomes, based on the available data. The same approach will be taken for dividing the treatment groups according to intensity, with the aim of observing the dose-response relationship.

#### Grouping for primary analysis comparisons

AVAT interventions and their comparison groups can be highly divers; therefore, we might group them into roughly similar intervention and comparison groups for the primary analysis, as indicated above. This will be done after the data extraction, but before data analysis, in order to minimise bias.

### Search strategy

Based on the recommendations from the Cochrane Handbook we will systematically search the Cochrane Library, EMBASE (via Ovid), and MEDLINE (via Ovid) [41]. Furthermore, we will search CINAHL, ERIC, APA PsycArticles, APA PsycInfo, and PSYNDEX (all via EBSCOHost), as well as the ClinicalTrials.gov and the WHO’s International Clinical Trials Registry Platform (ICTRP), which includes various smaller and national registries, such as the EU Clinical Trials Register and the German Clinical Trials Register (DRKS).

The search strategy is comprised of three search components; one concerning the art component, one the therapy component and the last consists of a recommended RCT filter for EMBASE, optimised for sensitivity and specificity [42–44]. See Additional file 2 for the complete search strategy, exemplified for the Cochrane Library search interface. In addition, relevant hand selected articles from individual databank searches, or studies identified through the screening of reference lists will be included in the review. A handsearch of The Journal of Creative Arts Therapies will be conducted.

Results of all languages will be considered, and efforts undertaken to translate articles wherever necessary. There will be no limitation regarding the date of publication of the studies.

### Data collection and data management

#### Study selection process

Two reviewers will independently scan and select the studies, first by title screening, second by abstract screening, and in a third step by full text reading. The two sets of identified studies will then be compared between the two researchers. In case of disagreement that cannot be resolved through discussion, a third researcher will be consulted to decide whether the study in question is eligible for inclusion. The Covidence software will be used for the study selection process [45].

### Data extraction

All relevant data concerning the outcomes, the participants, their condition, the intervention, the control group, the method of imputation of missing data, and the study design will be extracted by two researchers independently and then cross-checked, using a customised and piloted data extraction form. The chosen method of imputation for missing data (due to participant dropout or similar) will be extracted per outcome. Both, intention to treat (ITT) and per protocol (PP) data will be collected and analysed.

If crucial information will be missing from a study and its protocol, authors will be contacted for further details.

#### Risk of bias assessment for included studies

In line with the revised Cochrane risk of bias tool for randomised trials (RoB 2) [46], we will examine the internal bias in the included studies regarding their bias arising from the randomisation process, bias due to deviations from intended interventions, due to missing outcome data, bias in measurement of the outcome, and in selection of the reported result [47].

The risk will be assessed by two people independently from each other, only in cases of persisting disagreement a third person will be consulted.

If the final sample size allows, we will conduct an additional analysis in which the included studies are analysed separately by bias risk category.

#### Measures of treatment effect

If possible, we will conduct our main analyses using intention-to-treat data (ITT), but we will collect ITT and per-protocol (PP) data [48]. If for some studies ITT data is not reported, we will use the available PP data instead and perform a sensitivity analysis to see if that affects the results. Dichotomous data will be analysed using risk ratios with 95% confidence intervals, as they have been shown to be more intuitive to interpret than odds ratio for most people [49]. We will analyse continuous data using mean differences or standardised mean differences.

#### Unit of analysis issues

##### Cluster trials

If original studies did not account for a cluster design, a unit of analysis error may be present. In this case, we will use appropriate techniques to account for the cluster design. Studies in which the authors have adjusted the analysis for cluster-randomisation will be used directly.

##### Cross-over trials

An inherent risk to cross-over trials is the carry-over effect.

This design is also problematic when measuring unstable conditions such as psychotic episodes, as the timing could account more for the treatment success than the treatment itself (period effect).

As art therapy is used frequently in the treatment of unstable conditions, such as mental health problems or neurodegenerative disorders (i.e. Alzheimer’s), we will include full cross-over trials only if chronic and stable concepts are measured (such as permanent physical disabilities or epilepsy) [50].

When including cross-over studies measuring stable conditions, we will include both periods of the study. To incorporate the results into a meta-analysis we will combine means, SD or SE from both study periods and analyse them like a parallel group trial [51]. For bias assessment we will use the risk of bias tool for crossover trials [47].

For cross-over studies that measure unstable or degenerative conditions of interest, we will only include the first phase of the study as parallel group comparison to minimise the risk of carry-over or period effects. We will evaluate the risk of bias for those cross-over trials using the same standard risk of bias tool as for the parallel group randomised trials [52]. We will critically evaluate studies that analyse first period data separately, as this might be a form of selective reporting and the inclusion of this data might result in bias due to baseline differences. We might exclude studies that use this kind of two-stage analysis if we suspect selective reporting or high risk for baseline differences [47].

#### Missing data

Studies with a total dropout rate of over 50% will be excluded. To account for attrition bias, studies will be downrated in the risk of bias assessment (RoB 2 tool) if the dropout rate is more than half for either the control or the intervention group. An overall dropout rate of 25–50% we will also be downrated.

#### Assessment of clinical, methodological, and statistical heterogeneity

We will discuss the included studies before calculating statistical comparisons and group them into subgroups to assess their clinical and methodological heterogeneity. Statistical heterogeneity will be assessed by calculating the *p* value for the chi^2^ test. As few included studies may lead to insensitivity of the *p* value, we may adjust the cut-off of the *p* value if we only included a small amount of studies [49]. In addition, we will calculate the I^2^ statistic and its confidence interval, based on the chi^2^ statistic to assess statistical heterogeneity. We will explore possible reasons for observed heterogeneity, e.g. by conducting the planned subgroup analyses. Based on the amount and quality of included studies and their outcome heterogeneity, we will decide if a meta-analysis can be conducted. In case of high statistical heterogeneity, we first check for any potential errors during the data input stage of the review. In a second step, we evaluate if choosing a different effect measure, or if the justified removal of outliers will reduce heterogeneity. If the outcome heterogeneity of the selected studies is still too high, we will not conduct a meta-analysis. If clinical heterogeneity is high but can be reduced by adjusting our planned comparisons, we will do so.

#### Reporting bias

##### Funnel plot

Funnel plots can be a useful tool in detecting a possible publication bias. However, we are aware, that asymmetrical funnel plots can potentially have other causes than an underlying publication bias. As a certain number of studies is needed in order to create a meaningful funnel plot, we will only create those plots, if more than about 10 studies are included in the review.

### Data analysis and synthesis

Based on the amount and quality of included studies and their heterogeneity, we will decide if a meta-analysis is feasible.

If a meta-analysis can be conducted, we will be using the inverse variance method with random effects (to increase compatibility with the different identified effect measures and to account for the diversity of the included interventions). We would expect each study to measure a slightly different effect based on differing circumstances and differing intervention characteristics. Therefore, a random effects model is the most suitable option.

A disadvantage of the random effects model is that it does not give studies with large sample sizes enough weight when compared to studies with small sample sizes and therefore could lead to a small study effect. However, we expect to find studies with comparable study sizes with an *N* of 10–50, as very large trials are uncommon for art therapy research. If we include studies with a very large sample size, we might calculate a fixed effects model additionally, as sensitivity analysis, to assess if this would affect the results.

If the calculation of a meta-analysis is not advisable due to difficulties (such as a low number of included studies, low quality of included studies, high heterogeneity, incompletely reported outcome or effect estimates, differing effect measures that cannot be converted), we will choose the most appropriate method of narrative synthesis for our data, such as the ones described in the Cochrane Handbook (i.e. summarising effect estimates, combining *p* values or vote counting based on direction of effect) [53].

#### Subgroup analysis

If the number of included studies is large enough (around 10 or more [54]) and subgroups have an adequate size, we plan to compare subgroups based on the therapy setting (inpatient, outpatient, kind of institution), the intervention characteristics (the kind of AVAT, intensity of treatment, staff training, group size), the population (treatment indication, age, gender, country), or other study characteristics (e.g. bias category, publication date). If possible, we will also examine these factors by calculating meta-regressions.

#### Sensitivity analysis

Where possible, sensitivity analyses will be conducted using different methods to establish robustness of the overall results. Specifically, we will assess the robustness of the results regarding cluster randomisation and high risk of bias (RoB 2 tool).

## Discussion

AVAT encompasses a wide array of highly diverse treatment options for a multitude of treatment indications. Even though AVAT is a popular treatment method, the empirical base for its effectiveness is rather fragmented; many (often smaller) studies examined the effect of very specific kinds of AVATs, with a narrow focus on certain conditions [2, 7, 55, 56]. Our review will give a current overview over the entire field, with the hope of estimating the magnitude of its effectiveness. Several clinical guidelines recommend art therapy based solely on clinical consensus [57]. By accumulating all empirical evidence, this systematic review could inform the creation of future guidelines and thereby facilitate clinical decision-making.

Understanding the benefits, limits, and mechanisms of change of AVAT is crucial to optimally apply and tailor it to different contexts and settings. Consequently, by better understanding this intervention, we could potentially increase its effectiveness and optimise its application, which would lead to improved patient outcomes. This would not only benefit each individual who is treated with AVAT, but also the health care provider, who could apply the intervention in its most efficient way, thereby using their resources optimally.

Furthermore, explorative findings regarding the characteristics of the treatment could generate new hypotheses for future RCTs, for example regarding the effectiveness of certain types of AVAT for specific treatment indications. Moreover, the emergence of certain patterns in effectiveness could inspire further research about possible mechanisms of change of AVAT.

## Supplementary Information

**Additional file 1.** PRISMA-P checklist.

**Additional file 2.** Search strategy.

## Data Availability

The datasets used and/or analysed during the current study are available from the corresponding author on reasonable request.

## Abbreviations

AVAT: Active visual art therapy
PRISMA: Preferred Reporting Items for Systematic Reviews and Meta-Analyses
PRISMA-P: Preferred Reporting Items for Systematic Reviews and Meta-Analyses Protocols
RCT: Randomised controlled trial
RoB 2: Risk of Bias tool
ITT: Intention to treat
PP: Per protocol
