# Supplementary material for: The effect of active visual art therapy on health outcomes: protocol of a systematic review of randomised controlled trials
Source: Syst Rev. 2022 May 16;11:96. doi: 10.1186/s13643-022-01976-7 (PMC9109400; doi:10.1186/s13643-022-01976-7)
Supplement: Supplementary file 2 — Additional file 2. Search strategy. [file 13643_2022_1976_MOESM2_ESM.docx]

Search strategy for the Cochrane library

| ID | Search term |
| --- | --- |
|  | MeSH descriptor: [Art Therapy] explode all tre |
| #2 | MeSH descriptor: [Art] explode all trees |
| #3 | (art):ti,kw |
| #4 | (artist*):ti,kw |
| #5 | (artwork):ti,ab,kw |
| #6 | (creativ*):ti,kw |
| #7 | (Sketching*):ti,ab,kw |
| #8 | (coloring):ti,ab OR (colouring):ti,ab |
| #9 | (painting*):ti,kw |
| #10 | (doodle*):ti,ab,kw |
| #11 | (Craft*):ti,kw |
| #12 | (draw*):ti,kw |
| #13 | (printmaking):ti,kw |
| #14 | (“Fine art”):ti,ab,kw |
| #15 | ("sand painting"):ti,ab,kw |
| #16 | ("collage making"):ti,ab,kw |
| #17 | ("art on prescription"):ti,ab,kw |
| #18 | (sculpting):ti,kw |
| #19 | (mandala*):ti,ab,kw |
| #20 | ("clay work"):ti,ab,kw |
| #21 | ("bagh print*"):ti,ab,kw |
| #22 | (embroidery OR needlepoint OR Needlework):ti,ab,kw |
| #23 | ("lace-making"):ti,ab,kw |
| #24 | (patchwork):ti,ab,kw |
| #25 | ("pieced work"):ti,ab,kw |
| #26 | (patchwork):ti,ab,kw |
| #27 | ("pieced work"):ti,ab,kw |
| #28 | ("rug making"):ti,ab,kw |
| #29 | (lithography):ti,ab,kw |
| #30 | (weaving):ti,kw |
| #31 | (macramé OR makramee OR scoubidou OR crochet):ti,ab,kw |
| #32 | (knitting):ti,kw |
| #33 | (felting):ti,ab,kw |
| #34 | (“screen printing” OR silkscreening):ti,ab,kw |
| #35 | (tatting):ti,ab,kw |
| #36 | (woodworking OR "wood turning" OR "wood carving"):ti,ab,kw |
| #37 | (Beadwork):ti,ab,kw |
| #38 | ("glass etching"):ti,ab,kw |
| #39 | (tiffany):ti,kw |
| #40 | (glassblowing):ti,ab,kw |
| #41 | ("jewlery design" OR Silversmith*):ti,ab,kw |
| #42 | (goldsmith*):ti,kw |
| #43 | (pottery):ti,ab,kw |
| #44 | (mosaik):ti,ab,kw |
| #45 | (marquetry):ti,ab,kw |
| #46 | (bookbinding OR "card making" OR decoupage OR "embossing paper" OR "iris folding" OR origami OR "paper craft" OR "paper marbling" OR "paper modelling" OR "parchment craft" OR pergamano OR "paper filigree"):ti,ab,kw |
| #47 | ("Basket weaving" OR "doll* making" OR "flower arrangement" OR "floral design" OR "pressed flower craft" OR soapmaking OR "straw marquetry" OR "cake decorating" OR candlemaking OR "egg decorating"):ti,ab,kw |
| #48 | ("Contemporary art"):ti,ab,kw |
| #49 | ("Craft making" OR Handcraft* OR handicraft*):ti,kw |
| #50 | {OR #2-#49} |
| #51 | MeSH descriptor: [Psychotherapy] explode all trees |
| #52 | (therap*):ti,kw |
| #53 | MeSH descriptor: [Health] explode all trees |
| #54 | MeSH descriptor: [Mental Health] explode all trees |
| #55 | (intervention):ti,kw |
| #56 | (prevent*):ti,kw |
| #57 | (Palliative):ti,kw |
| #58 | (prevent*):ti,kw |
| #59 | (rehabilita*):ti,kw |
| #60 | MeSH descriptor: [Nursing] explode all trees |
| #61 | (recovery):ti,kw |
| #62 | (healing):ti,kw |
| #63 | {or #51-#62} |
| #64 | (#50 AND #63) |
| #65 | (#1 OR #64) |
| #66 | (antiretroviral):ti,ab,kw OR (anti-retroviral):ti,ab,kw |
| #67 | MeSH descriptor: [Anti-Retroviral Agents] explode all trees |
| #68 | (assisted reproductive technology):ti,kw |
| #69 | ("state of the art"):ti,kw |
| #70 | ("State-of-the-art"):ti,kw |
| #71 | {OR #66-#70} |
| #72 | (#65 NOT #71) |
